# Scoliosis and dental occlusion: a review of the literature

**DOI:** 10.1186/1748-7161-6-15

**Published:** 2011-07-29

**Authors:** Matteo Saccucci, Lucia Tettamanti, Stefano Mummolo, Antonella Polimeni, Felice Festa, Simona Tecco

**Affiliations:** 1 Department of Oral Science, University La Sapienza, Via Caserta 6, 00161, Rome, Italy; 2 Department of Surgical Reconstructive Sciences and Advanced Technologie, University of Insubria, Via G. Piatti, 10 - 21100 Velate, Varese, Italy; 3 Department of Health Science, University of L'Aquila, Edificio Delta 6 L'Aquila Fraz. Coppito, 67010, L'Aquila, Italy; 4 Department of Oral Science, Nano and Biotechnology, University G.D'Annunzio, Via dei Vestini 31, 66013, Chieti, Italy

## Abstract

**Background:**

Idiopathic scoliosis is a deformity without clear etiology. It is unclear wether there is an association between malocclusion and scoliosis. Several types of occlusion were described in subjects with scoliosis, mostly case-reports.

**Objectives:**

The aim of this review was to evaluate the type of occluslins more prevalent in subjects with scoliosis

**Search strategy:**

All randomised and controlled clinical trials identified from the Cochrane Oral Health Group Trials Register, a MEDLINE search using the Mesh term scoliosis, malocclusion, and relevant free text words, and the bibliographies of papers and review articles which reported the outcome of orthodontic treatment in subjects with scoliosis that were published as abstracts or papers between 1970 and 2010.

**Selection criteria:**

All randomised and controlled clinical trials published as full papers or abstracts which reported quantitative data on the outcomes malocclusion in subjects with scoliosis.

**Data collection and analysis:**

Data were extracted without blinding to the authors, age of patients or type of occlusion.

**Main results:**

Using the search strategy eleven observational longitudinal studies were identified. No randomized clinical trials were recorded. Twenty-three cross-sectional studies were recorderd, and the others studies were reviews, editorials, case-reports, or opinions. The clinical trials were often not controlled and were about the cephalometric evaluation after treatment with the modified Milwuakee brace, followed by the orthodontic treatment of the class II relationship with a functional appliance. Clinical trials also included the study of the associations between scoliosis and unilateral crossbite, in children with asymmetry of the upper cervical spine. This association was also investigated in rats, pigs and rabbits in clinical trials. The other associations between scoliosis and occlusion seems to be based only on cross-sectional studies, case-reports, opinions.

**Authors' conclusions:**

Based on selected studies, this review concludes that there is plausible evidence for an increased prevalence of unilateral Angle Class II malocclusions associated with scoliosis, and an increased risk of lateral crossbite, midline deviation in children affected by scoliosis. Also, documentation of associations between reduced range of lateral movements and scoliosis seem convincing. Data are also mentioned about the association between plagiocephaly and scoliosis.

## Introduction

Idiopathic scoliosis is a deformity without clear etiology. Depending on the age of presentation it has been classified into 3 types: infantile (presenting from birth to 3 years), juvenile (presenting from 3 to 10 years) and adolescent (presenting from 10 years to skeletal maturity) [1]. Eighty percent or more of idiopathic scoliosis is of the adolescent variety [2]. The most infantile curves present in the first six months of life are left thoracic apex, and males are more frequently affected, whereas the most common juvenile curves are right thoracic apex and females are more frequently affected, as in the adolescent group [3]

In the case of the most common form of scoliosis, adolescent idiopathic scoliosis, there is no clear causal agent and it is generally believed to be multifactorial. Genetics are believed to play a role [4]. There is often a positive family history but the pattern of inherited susceptibility is not clear [5]. Adolescent idiopathic scoliosis is defined as a spinal curve or curves of ten degrees or more in about 2.5% of most populations [5]. However, in only about 0.25% the curve does progress to the point that treatment is warranted [5].

Some hypothesis exists in the possible underlying pathophysiological mechanism leading to this deformity. The major types of non-idiopathic scoliosis are congenital scoliosis due to malformation or faulty segmentation of the vertebrae and neuromuscular scoliosis due to muscular imbalance.

The scoliosis can be due to malformation or faulty segmentation of the vertebrae or can be due to muscular imbalance [1].

Different factors have been suggested as causal. Among these, the following should be highlighted: deviation from the standard growth pattern, neuromuscular or conjunctive tissue alterations, asymmetric growth of the limbs and trunk, alterations in the sagittal configuration of the spine; and environmental factors [6,7].

Non-congenital scoliosis has many etiologies. The hereditary musculoskeletal disorders, such as osteogenesis imperfecta, Marfan syndrome, Stickler syndrome, Ehlers-Danlos syndrome, and the muscular dystrophies, can each include scoliosis as a manifestation. Neuromuscular diseases, such as cerebral palsy and myelomeningocele, are associated with the development of scoliosis secondary to muscle imbalance. Paralytic disorders resulting from polio or spinal trauma may lead to a progressive scoliosis [1].

In dentistry, the study of the relationship between occlusal problems and the spine are of increasing interest. This is the result of a greater incidence of pain in the muscles of the neck, trunk, the upper and lower limbs, and in the temporomandibular joints (TMJ) of patients with occlusal dysfunction [5]. There are several conditions that impede normal trunk alignment in the frontal plane, and it appear interesting to investigate whether such conditions also affect dental occlusion.

Since '70 Fonder, [8] a dentist, presented case history evidence to evidence a causal relationship between occlusion and scoliosis, and vice-versa, as he underlined the relationship of dental malocclusions to various skeletal problems such as scoliosis, kyphosis, and other postural defects. He showed full spine radiographs, both lateral and frontal, before and after dental treatment for malocclusions in three patients. Case I exhibited noticeable scoliosis and other "defects of posture" notably excess thoracic kyphosis, in the pre-treatment films. Following a course of dental treatment for a bite defect, the post-treatment radiograph revealed a non scoliotic spine with normal lateral and antero-posterior curvatures. Case 2 was similar except that the scoliosis and kyphosis before the treatment were less marked, described as being only a case of bad posture. After orthodontic treatment for deep overbite related to posterior malocclusion, the post-treatment x-ray revealed a normal appearing spine. Fonder described the patient as having greatly improved posture. In the third case, a woman with similar abnormal scoliotic and kyphotic curves in the spine also complained of general ill health with headaches, backaches and limited range of motion of the back. Following prosthetic and other standard dental work, all of these symptoms were said to disappear and the spine on post-treatment x-ray examination appeared more normal.

The purpose of this review is to summarize what is known about the data in literature regarding the association of scoliosis with altered teeth occlusion, hereditated or acquired, and possibly to evidence the natural history of idiopathic scoliosis after the malocclusion treatment, as well as the long term effects of treatment, if investigated (Table 1).

**Table 1:** Principal papers showed in this review.

Paper	Type	Main topic	Sample	Age	Main result
McMaster J (1965) Reference [7]	3 clinical cases	Casual relationship between malocclusion and scoliosis, and viceversa	3 adolescents	10-15 years	After orthodontic treatment, the author observed the improvment of posture

**Paper**	**Type**	**Main topic**	**Sample**	**Age**	**Main result**

Rock and Baker (1972). Reference [9]	Case-report	Class II due to the weared cast	A girl	14 years old	to recommend the use of a removable appliance to prevent the malocclusion before the surgeon operation and during the period of the wearing the cast.

**Paper**	**Type**	**Main topic**	**Sample**	**Age**	**Main result**

Dayan et al. (1977). Reference [10]	Transversal Case-control study	To compare facial morphology of children affected with scoliosis and treated with brace, with health children	15	5-19 years (mean 10 years)	Children treated with braces (for their scoliosis) showed all vertical measurements of face significantly lower than the control group, and more protruted maxillary and mandibular bases

**Paper**	**Type**	**Main topic**	**Sample**	**Age**	**Main result**

Hotchcock HP (1969). Reference [8]	Observational study on prevalence	Plagiocephaly in subjects with scoliosis	144		The study suggested the existence of an association between infantile scoliosis and plagiocephaly

**Paper**	**Type**	**Main topic**	**Sample**	**Age**	**Main result**

Ben-Bassat Y et al. (2006) Reference [16]	Observational study on prevalence	Prevalence of scoliosis in patients with ereditated malocclusion	202 adolescents	10-15	The detection of hereditary orthodontic anomalies in young children allows the identification of a group of children who have a high risk of developing scoliosis in later years.
**Paper**	**Type**	**Main topic**	**Sample**	**Age**	**Main result**
Segatto et al. (2008) Reference [17]	Cohort study	Malocclusion in subjects with idiopathic scoliosis	98 subjects with scoliosis and 705 controls	6.2 - 25.3; mean age 13.9 +/- 3.5	a significant higher prevalence of unilateral Angle class II (asymmetric class II malocclusion) was evident among the patients with scoliosis (21.9%) compared with the control group (8.5%). The differences between the two groups in the prevalence of the midline deviation were statistically significant both in the upper and the lower dental arches.

**Paper**	**Type**	**Main topic**	**Sample**	**Age**	**Main result**

**Lippold C et al. (2003)**. **Reference **[18]	**Case-control study**	**To evaluate the differences in occlusion**	**28 with scoliosis and 68 health children**	**Mean age 14.7 +/- 2.3**	In the group of adolescents with scoliosis, infacts, the unilateral Angle class II relationship showed a significant higher prevalence respect to the control group

**Paper**	**Type**	**Main topic**	**Sample**	**Age**	**Main result**

**Lippold et al. (2007)** **Reference **[19]	**Observational**	**To compare**	**53 adult patients with Class II and Class III, but withut scoliosis**	**24.6 +/- 9**	an orthopedic examination can be considered for patients undergoing an orthodontic-operative therapy, also when they don't show scoliosis.

**Paper**	**Type**	**Main topic**	**Sample**	**Age**	**Main result**

**Korbmacher H. et al. (2007)**. **Reference **[22]	**Case-control study**	**Prevalence of scoliosis in subjects with jaw deformity**	**85 patients with jaw deformity and 20 control subjects**	**adults**	Of the 85 patients with jaw deformity, 23 (27.1%) had a Cobb angle exceeding 10°. None of the control group had scoliosis exceeding 10°.

**Paper**	**Type**	**Main topic**	**Sample**	**Age**	**Main result**

**Pedrotti et al. (2007)**. **Reference **[23]	**Case-control**	**To assess the congruence of the laterality of cross-bite and the orthopaedic asymmetry**	**55 children with unilateral cross-bite, and 55 children with asymmetric cervical spine (and no cross-bite)**	**3-10**	among the children who revealed an asymmetric upper cervical spine, the unilateral crossbite was not necessarily combined with a pathological orthopaedic variable,

**Paper**	**Type**	**Main topic**	**Sample**	**Age**	**Main result**

**Lippold et al. (2000)** **Reference **[24]	**A prevalence stuydy**	**Prevalence of bilateral crossbite in subjects with scoliosis**	**428**	**9-14**	an incidence of scoliotic attitudes of 9.5%, with a statistically significant relationship among that disorders of posture, and the presence of ogival palate with bilateral crossbite

**Paper**	**Type**	**Main topic**	**Sample**	**Age**	**Main result**

**Azuma Y et al. (1999);** **D'Attilio M et al. (2005);** **Poikela A et al. (1997);** **Nerder PH et al. (1999)**. **References **[34-37]	**Animal studies**	**The appearence of scoliosis after an imbalance of occlusion**	**animals**	**/**	these experimental studies revealed a high level of asymmetry in craniofacial structures, temporomandibular structures and muscle functions after an experimentally induced crossbite

## Objectives

### Primary objective

Our primary objective was to systematically review the literature to determine the incidence of malocclusion in adult and adolescents with scoliosis.

We did not consider other postural orthopaedic problems since scoliosis is a well defined pathology in literature.

### Secondary objectives

Our secondary objectives were to

1) Assess the clinical consequences for the malocclusion, after the treatment of scoliosis (clinical symptoms).

2) Assess the clinical consequences for the scoliosis, after the orthodontic treatment (clinical complications and symptoms associated with scoliosis, and severity of complications and symptoms among patients).

## Methods

Criteria for considering studies for this review

### Types of studies

We looked for randomized clinical trials (RCTs), cohort and case-control studies, and case reports.

### Types of patients

We included adolescent subjects with malocclusion and scoliosis. For our secondary objective we included patients if they were children/adolescents treated for their scoliosis or malocclusion.

### Type of studies

We included studies that reported incidence and desciption of malocclusion associated with scoliosis.

### Types of outcomes

Our primary and first secondary outcome of interest was incidence and description of malocclusion in subjects with scoliosis.

Our secondary outcomes of interest were the clinical consequences associated with treatments of malocclusions or scoliosis.

### Electronic searches and data retrieval

We searched MEDLINE and EMBASE without language restrictions in September 2010. We also manually searched reference lists from recent review articles. All randomised and controlled clinical trials identified from the Cochrane Oral Health Group Trials Register, a MEDLINE search using the Mesh term scoliosis, malocclusion, and relevant free text words, handsearching the British, European and American journals of orthodontics and Angle Orthodontist, and the bibliographies of papers and review articles which reported the outcome of orthodontic treatment in subjects with scoliosis that were published as abstracts or papers between 1970 and 2010.

### Study selection and Data Extractiones of interventions

Two reviewers (ST and MS) independently reviewed the abstracts for potential eligibility and subsequently full text publications for eligibility. Disagreements were resolved by discussion.

We extracted a number of variables on study design and methodological characteristics, patient and intervention characteristics, and outcomes from all eligible studies (see Table 1). Data extraction was done independently by two reviewers (ST and MS) and disagreements were resolved by discussion.

### Methodological Quality Assessment

No Randomized clinical trials were recorded for this argument.

For the observational longitudinal studies we noted the presence of control groups only in a few studies. The great part of transversal studies showed a control group.

## Results

Using the search strategy eleven observational longitudinal studies were identified. No randomized clinical trials were recorded. Twenty-three cross-sectional studies were recorderd, and the others studies was reviews, editorials, case-reports, or opinions. The clinical trials were often not controlled and were about the cephalometric evaluation after treatment with the modified Milwuakee brace, followed by the orthodontic treatment of the class II relationship with a functional appliance. Clinical trials also included the study of the associations between scoliosis and unilateral crossbite, in children with asymmetry of the upper cervical spine. This association was also investigated in rats, pigs and rabbits in clinical trials. The other associations between scoliosis and occlusion seems to be based only on cross-sectional studies, case-reports, opinions.

### Scoliosis and Plagiocephaly

In literature, the association between plagyocephaly and scoliosis was observed during '80 decade in premature infants. The existence of an association is based on clinical case-reports, opinins and cross-sectional studies.

**Figure 1:**
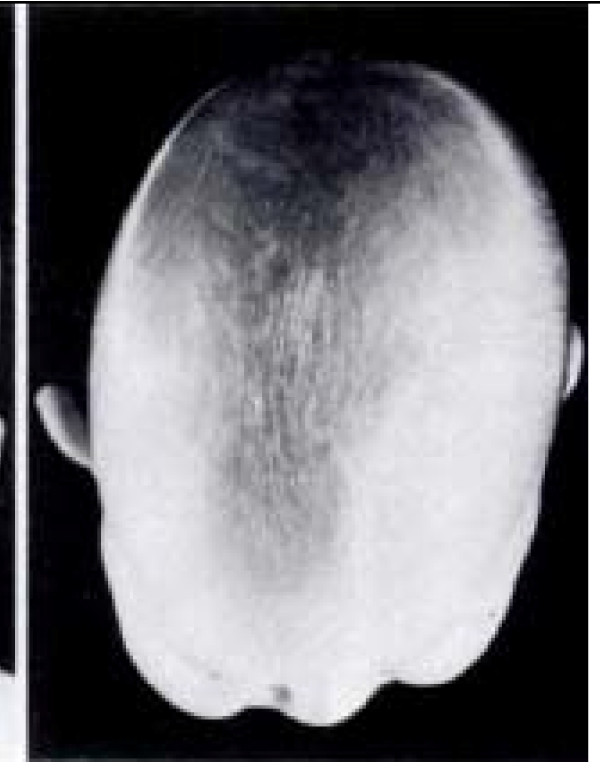
**Left sided plagiocephaly with controlateral bat ear**. Tracted by the paper referenced in [3].

In a study performed on 144 infantile patients who attended the Edinburgh Scoliosis Clinic between 1968 and 1982, [9] plagiocephaly was present in 124 infants (86%) and absent in nine (6%), all with resolving curves; no clinical records had been made in the remaining 11 infants (8%). In the patients with progressive curves, either single or double curves, the "recessed" side of the plagiocephaly always corresponded with the convex side of the thoracic or thoracolumbar curve, suggesting the existence of an association between infantile scoliosis and plagiocephaly [8]. (Figure 1)

Also in the patients affected by scoliosis and plagiocephaly, who showed a resolving scoliotic curve, the "recessed" side of the head corresponded with the convex side of the curve [8,9].

The association between these two conditions has been explained by the nature of plagiocephaly, that is a plastic deformation of the skull. It was hypothesized that when an immobile infant habitually lies towards one side (the case of premature babies) the action of gravity on the plastic skull could cause the uppermost side of the face and head to flow backwards and become recessed, while the lower ear is pushed forwards producing the commonly associated contralateral "bat ear". Associated with this immobility, plagiocephaly, however, rarely persists and once the child becomes mobile, it usually resolves by the age of six years. The scoliosis in these infants was rarely noted at birth but, like the plagiocephaly, developed within the first six months of life in 70% of subjects. In the cited sample, the convex side of the curve corresponded with the recessed side of the head in all except four infants with resolving curves. This close association between the presence, time of presentation and side of the two deformities (both plagiocephaly and infantile idiopathic scoliosis) suggested a possible common pathogenesis.

### The Milwuakee brace and malocclusion

A lot of studies made on 60' and 70' years on the use of the original Milwuakee brace in scoliosis therapy demonstrated the damageable effects on teeth occlusion.

About this argument, longitudinal clinical trials were recorded, in addition to clinical cases and observational studies.

**Figure 2:**
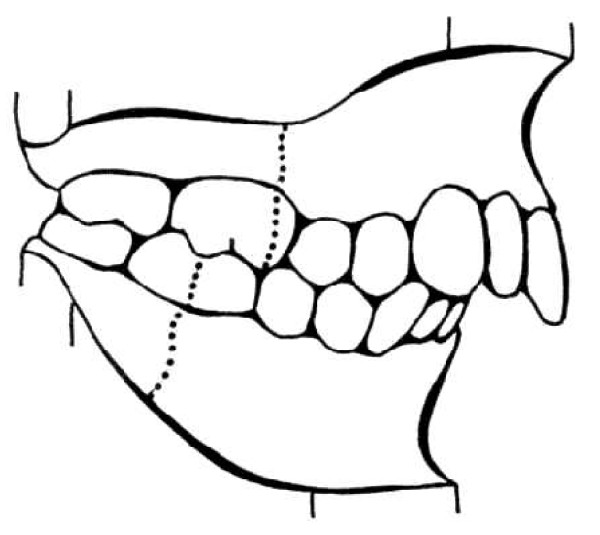
**Angle Class II molar relationship**. For the malocclusion to satisfy the definition of a full-step Class II molar relationship, the mesiobuccal cusp of the maxillary permanent first molar must occlude, at least on one side, in the embrasure between the mandibular second premolar and the mandibular permanent first molar, or farther to the mesial. If the maxillary or mandibular permanent first molar is missing, the buccal cusp of the maxillary second premolar must occlude in the embrasure between the mandibular first and second premolars, or farther to the mesial. If the maxillary permanent first molar has drifted mesially due to premature loss of the deciduous second molar, that is not considered a full-step Class II molar relationship.

**Figure 3:**
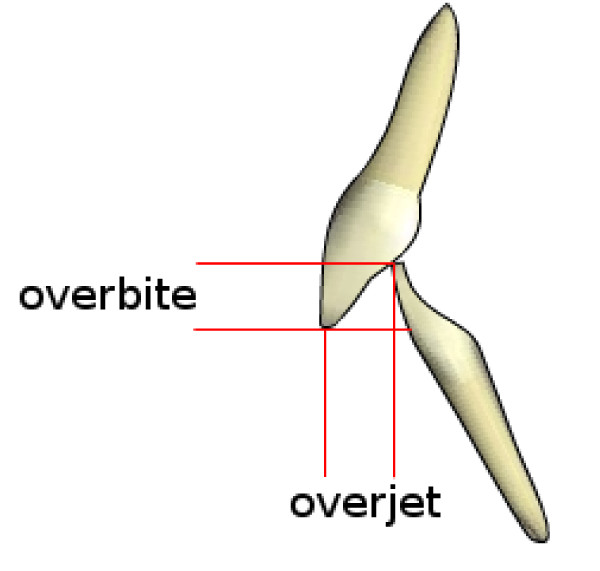
**Overjet and overbite**.

**Figure 4:**
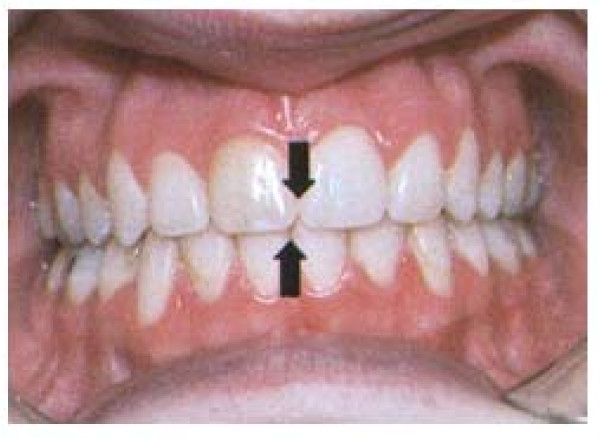
**Dental midline deviation**. Tracted by the paper referenced in [52].

**Figure 5:**
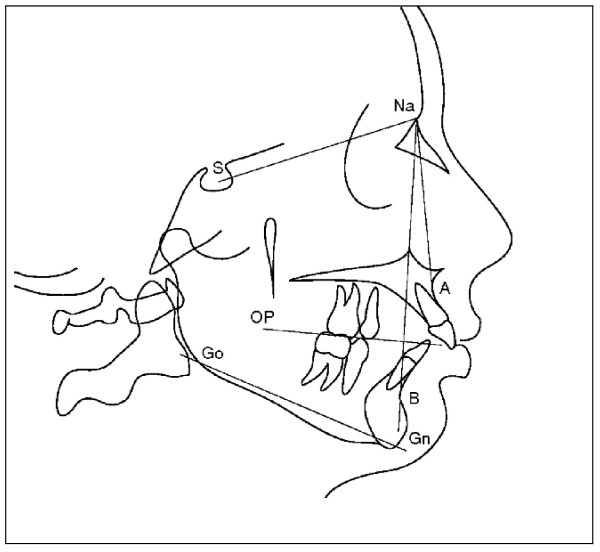
**Protrusion of maxilla - SNA angle - and retrusion of manibula - SNB angle - in a cephalometric tracing**. OP: Occlusal plane; GoGn: Mandibular plane. Tracted by the paper referenced in [16].

**Figure 6:**
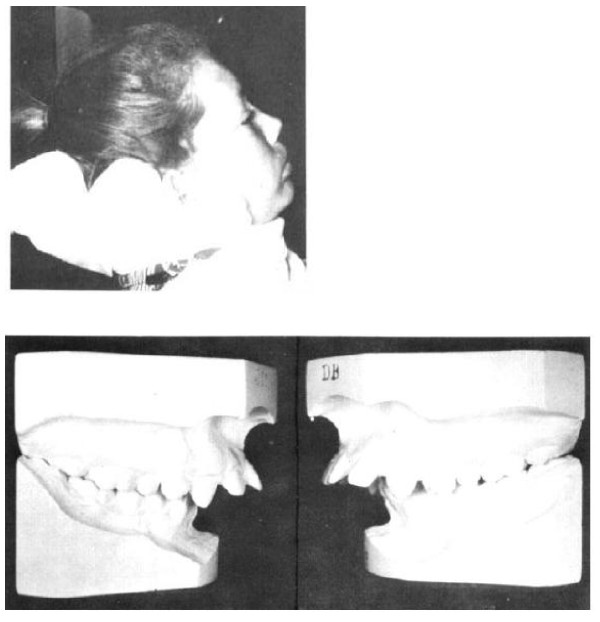
**a-b**. (a)The cast was relieved under the chin. (b) The class II malocclusion associated to the cast. Tracted by the paper referenced [9].

In 1969, a clinical case was published about the orthodontic treatment of a class II malocclusion in a patient, probably caused by the cast worn after the surgeon operation to reduce the scoliosis, in a fourteen year old girl who received the Harrington operation in 1963 [10]. This article suggest the great attention given to the association between class II malocclusion (Figures 2, 3, 4 and 5) and the use of this type of orthopedic brace, due to the presence of a force on the chin (Figure 6); the correction of the class II malocclusion requested the wearing of only a removable appliance (a positioner). The patient was treated soon after the operation and, for a year later, with a positioner, after which the malocclusion resulted corrected. For this, the conclusion of that article was to recommend the use of this removable appliance to prevent the malocclusion before the surgeon operation and during the period of wearing the cast.

**Figure 7:**
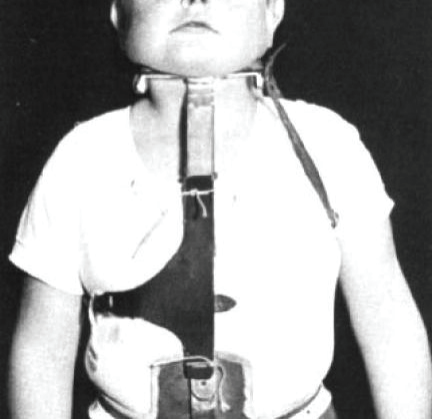
**A patient wearing the Milwuakee brace**. Tracted by the paper referenced in [9].

**Figure 8:**
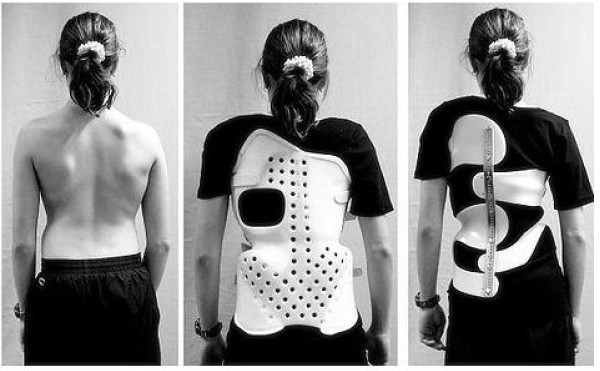
**Orthopaedic braces used today**. Tracted by the paper referenced in [15].

In the April of 1972, the effect of the Milwaukee brace upon dentofacial growth was investigated in a longitudinal clinical trial in a large sample and compared with a control group [11]. Measurements of facial morphology at different age of subjects affected by scoliosis and treated with this device, were compared of a health matched sample. The age range was about 5 to 19 years, with a mean of 10 years. The differences were associated to the wearing of the brace (Figure 7). All vertical measurements of face were significantly lower than the control group. The mandible and the maxilla were significantly more protruted in the study group than in the control one. The suggestion was to wear a teeth positioner during the therapy. However, as no pre-treatment data were available, it is not sure that these characteristics were caused by the brace, although it is evident that the brace can reduce the vertical dimension of the face. The Milwaukee brace has undergone many modifications since its creation. The chin pad in the original brace was replaced by a plastic throat piece in a lower position and closer to the neck. In this new design, its posture was underneath the body of the mandible just above the thyroid cartilage, so that the patient would not be able to rest the mandible on the throat piece, as was previously done with the chin pad. The rigid occipital pad was changed into flexible plastic uprights to allow the patient to tip his head backwards. This modified brace was more comfortable to wear with less pressure under the mandible. Also the use of a removable splint was suggested to avoid dental consequences when the patient did not show permanent teeth, and wore the brace for more than 24 months [12]. The effect of an orthodontic device was also evidenced in clinical case-reports [13,14]. Bracing is normally done when the patient has bone growth remaining and is generally implemented to hold the curve and prevent it from progressing to the point where surgery is recommended. Braces are also sometimes prescribed for adults to relieve pain. Bracing involves fitting the patient with a device that covers the torso; in some cases it extends to the neck. Today, the most commonly used brace is a TLSO, a corset-like appliance (Figure 8) that fits from armpits to hips and is custom-made from fiberglass or plastic. It is usually worn 22-23 hours a day and applies pressure on the curves in the spine. The effectiveness of the brace depends not only on brace design and orthotist skill, but on patient compliance and amount of wear per day. The latest standard of brace construction is with CAD/CAM technology. With the help of this technology it has been possible to standardize the pattern specific brace treatment. Severe mistakes in brace construction are largely ruled out with the help of these systems. A more recent development is the SpineCor Dynamic brace. It was developed by a research team at the St. Justine Hospital in Montreal Canada, as part of a research project funded by the Canadian government. The brace was first used in clinical application in Montreal in 1992 and is currently used in many countries throughout the world. This brace works using a different treatment approach to rigid bracing. Rather than trying to force the spine straight using three points of pressure, SpineCor uses a corrective movement. The regions of the body -- shoulders, rib cage, lumbar spine and pelvis -- are guided to a postural position that is the inverse of the scoliotic posture. As the spine is connected to the body it must move with the body when it is repositioned by the corrective movement. Hence, through the coupling of postural and spinal position, it is possible to affect the geometry of the scoliotic curve. The advantages of SpineCor are that it is flexible and allows dynamic movement, thereby eliminating the muscle weakening side effects seen with rigid bracing. It is also very easily concealed under clothing. The fact that it works as both a rehabilitation device and a brace, means that corrections made in the brace are sustained over the long term in 95.7% of cases [15]. In view of the postural approach to correct the scoliosis, the contemporary correction of occlusal deviations can be considered in line with the actual principles, based on the postural correction, of scoliotic treatment. Typically, braces are used for idiopathic curves that are not grave enough to warrant surgery, but they may also be used to prevent the progression of more severe curves in young children, to buy the child time to grow before performing surgery, which would prevent further growth in the part of the spine affected. The Scoliosis Research Society's recommendations for bracing include curves progressing to larger than 25 degrees, curves presenting between 30 and 45 degrees, Risser Sign 0, 1, or 2 (an x-ray measurement of a pelvic growth area), and less than 6 months from the onset of menses in girls [16].

### *Scoliosis and Angle class II molar relationship *(unilateral class II malocclusion)

This relationship was investigated mostly through case-control studies and clinical case reports. Among the orthodontic problems associated with scoliosis, attention was given to hereditary orthodontic anomalies (class III, crowding, ogival palate). Hereditary orthodontic anomalies were found at a significant level in a group of 202 adolescent patients diagnosed with idiopathic scoliosis, with a Cobb angle from 20° to 50°, [17] compared with a matched control health group, while acquired orthodontic anomalies occurred in both groups at about the same rate of frequency, suggesting that the detection of hereditary orthodontic anomalies in young children allows the identification of a group of children who have a high risk of developing scoliosis in later years.

In 2006, the occlusions (Figure 1,2, 3, 4 and 5) of patients with idiopathic scoliosis were clinically examined in a group of 96 consecutive orthopedic patients with idiopathic scoliosis (79 females and 17 males: mean age, 13.9 y; SD: 3.5 y; range, 6.2-25.3 y) [18]. Occlusal features of a random group of 705 children served as the control. In the considered sample, the interarch relationships in the antero-posterior dimension (Angle classification) were similar in the 2 groups for the frequency distributions for normocclusion and Class I malocclusion, but they were significantly different when concerned the Angle class II malocclusion (Figure 2, 3 and 5).

The distribution of the Angle class II malocclusion was significantly different in the scoliotic patients respect to the orthopedic health group.

Specifically, taking in consideration the group of subjects with Class II malocclusion, (Figure 2) with a high overjet (Figure 3), a significant higher prevalence of unilateral Angle class II (asymmetric class II malocclusion) was evident among the patients with scoliosis (21.9%) compared with the control group (8.5%), indicating that the asymmetry in the antero-posterior relationships seems a clinical sign associated with scoliosis.

In particular, while the frequency of asymmetrical molar relationships was identical in the scoliosis and the control groups, great differences in the frequency of asymmetrical canine relationships were encountered; the scoliosis patients were more asymmetric in this regard. In addition, in the same sample, the prevalence of upper midline deviation (Figure 4) (this is a clear clinical sign of occlusal asymmetry) was 21% in the group of scoliosis and 9.5% in the control group; at the same time, the prevalence of lower midline deviation was 53.7% in the study group and 32.9% in the control group. The differences between the two groups in the prevalence of the midline deviation were statistically significant both in the upper and the lower dental arches. No association was found between site, side, or severity of scoliosis and the appearance or site of the malocclusion features examined.

Later, the severity of scoliosis was related to the occlusal relationship again, but no significant relationship was observed between the severity of scoliosis and the occlusal characteristics.

In 2008, in facts, the scoliosis was related again to the Angle class malocclusion, [19] with the analysis of 28 children with scoliosis at various degree of severity (mean age: 14.7 y; SD: 2.3 y) matched with a control group of 68 orthopedically healthy children (mean age: 14.8 y; SD: 0.11 y). In the group of scoliotic subjects, the indication of the corset was represented by the values of Cobb angle > 20° measured at the level of main curvature, so these children belonged to the severe group. In the analyzed sample, nine children were wearing corset because of the severity of their orthopedic malformation. The moderate subgroup consisted of children with malformations requiring no constant posture correction, namely wearing corset. Besides the clarified different orthopedic situation, the selection criteria of the two groups were: similar age, no previous orthodontic treatment, as well as no missing teeth, carious lesions, or pathologic periodontal status. In this sample, when analyzing the sagittal deviations in the molar region, the incidence rate of the bilateral deviation, being present as a sign of symmetry, as well as of the unilateral occlusal deviation, related to the asymmetry, revealed a significant importance. In the group of adolescents with scoliosis, in fact, the unilateral Angle class II relationship showed a significant higher prevalence respect to the control group (Figure 2). Specifically, in the group of subjects with scoliosis, the 57.12% showed a normal bilateral occlusion, but the 28.56% showed a unilateral Angle class II malocclusion, with a significant higher frequency respect to the health group. This unilateral deviation (unilateral Angle class II malocclusion) was characteristic for almost one-third of scoliotic subjects, while in the control health group its incidence rate was hardly 8.82%. In the group of scoliotic subjects, the unilateral class II relationship was significantly higher frequent then the bilateral class II relationship, pointing on the importance of the asymmetry of malocclusion, in relation to the scoliosis.

These studies are in accordance with what affirmed by Lippold et al. (2003), [20] that the scoliotic curves occur in the frontal plane and - through the head posture that is tilted sideways -play an important role in the development of the different dentofacial asymmetries. Results of several studies, as seen, confirm a potential correlation between scoliosis and unilateral Class II malocclusion.

Unilateral Class II malocclusion is not the only type of malocclusion significantly associated to the scoliosis.

Segatto *et al*. (2008) [18] analyzed also other occlusal characteristics of the frontal region of dental arch and found some other significant differences between the scoliotic and the health groups.

In particular, the subjects with scoliosis showed a significant higher overjet (see Figure 3 for details on this variable) and a higher midline deviation (Figure 4) (Table 2) respect to the control group. Then, the scoliotic group was characterized by lower overbite (Figure 3 for details on this variable) compared to the determined mean values (3.10 mm) of the control health group (Table 3) [18].

**Table 2:** Frequency of the sagittal occlusal anomalies on the molar region, according to the study by Segatto et al. (2008).

Parameters		Scoliosis group	Control group
Normal molar occlusion (Angle Cl.I) frequency (%) (health occlusion)	unilateral	**28.56**	**16.17**
	bilateral	57.12	64.68
Distal molar occlusion (Angle Cl.II) frequency(%) (pathological occlusion)	unilateral	**28.56**	**8.82**
	bilateral	10.07	16.17

**Table 3:** Comparison of the occlusal characteristics of the frontal region, according to the study by Segatto et al. (2008).

Parameters	Scoliosis group	Control group	
	**severe type**	**moderate type**	
**Overjet**			
mean ± SD (mm)	2.74 ± 1.851	2.55 ± 1.509	2.21 ± 1.201
**Overbite**			
mean ± SD (mm)	2.58 ± 2.168	2.78 ± 1.715	3.10 ± 1.585
**Midline deviation**			
mean ± SD (mm)	2.08 ± 1.121	1.76 ± 0.972	1.47 ± 0.898

Finally, on the basis of the evaluation of cephalograms in the same sample, a slightly protrusive maxilla and a slightly retrusive mandibula (Figure 5), characteristic of a class II skeletal discrepancy, resulted more pronounced in the scoliotic group than in the control group [19].

In addition to the studies that compared scoliotic to healthy subjects, other investigations underlined a relation between the occlusion and the vertebral column alignment, also in not scoliotic subjects [20,21].

These studies thus suggest a multidisciplinary orthodontic and orthopaedic approach to patients who do not show any clinical evidence of scoliosis or malocclusion.

**Figure 9:**
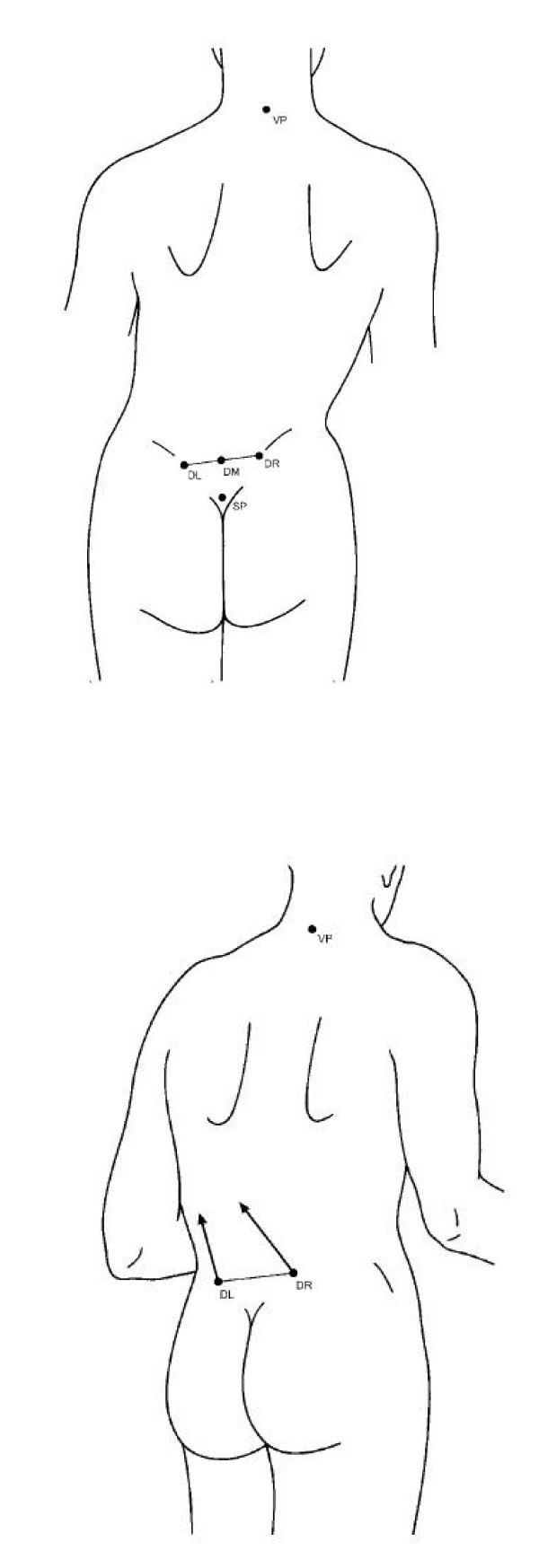
**a-b. (a)Pelvic tilt: the difference in height between the DR and the DL (right crista iliaca posterior superior [DR], and left crista iliaca posterior superior [DL]) measured in millimeters**. The angle between the vertical passing through DR and DL to the horizontal reference plane was defined as angular measure in degrees. (b) Pelvic torsion was measured by the angle between the surface normals to the lumbar dimples indicating the spina iliaca posterior superior landmark. In a symmetric pelvis without torsion of the iliac bones, pelvic torsion angle is 0. The angle is positive if the normal to the right dimple points lower than the normal to the left dimple, indicating the DR to be rotated backward whereas the DL is rotated forward. Tracted by the paper referenced in [11].

For example, in 2007, Lippold et al. noted [21] a relationship between the pelvic tilt and pelvic torsion (Figure 9a-b) and the facial shape (facial axis and facial depth), variables which affect the occlusion of teeth and influence the orthodontic treatment.

The study was performed on a group of fifty-three adult patients (32 women and 21 men; mean age 24.6 years, SD 9.0 years) with skeletal malformations (Class II and III malocclusion) who came to medical center for a consultation regarding an orthodontic treatment, without anamnestically established motor or neurological findings and/or previous internal or orthopaedic illnesses.

In the sample, some correlations were observed with the facial depth (mesial/distal) and the facial axis (vertical/horizontal).

Patients with a vertical value on the facial axis and a skeletal distal value in the facial depth (long face) had a slight pelvic torsion (Figure 9b) where the DL (left crista iliaca posterior superior) was rotated backward with respect to the DR (right crista iliaca posterior superior), while patients with a horizontal facial axis and mesial relation of facial depth (short face) revealed a slight rotation of the DR rotated backward regarding the contralateral side. Although the investigation was performed on subjects without a diagnosed scoliosis, and on the base of a rasterstereographic surface reconstruction of the back profile of a patient (and not a radiographic evaluation of scoliosis), it suggested an extension of the interdisciplinary concepts within the sense that an orthopedic examination can be considered for patients undergoing an orthodontic-operative therapy.

### Scoliosis and crossbite (Figure 10)

**Figure 10:**
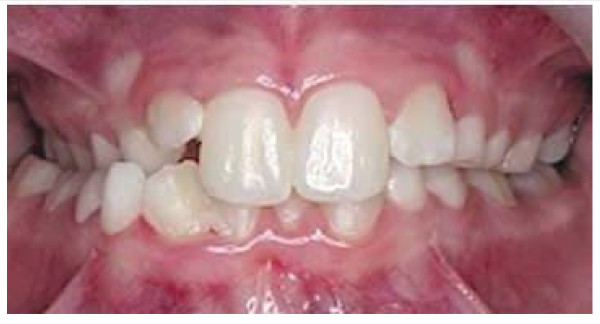
**Lateral crossbite in the right side of the patient**. In the left side, the occlusion is normal. Tracted by the paper referenced in [43].

This relationship was investigated mostly through case-control studies and clinical case-reports. In general, it was stated that left-right asymmetries are among the most common anomalies in patients with scoliosis [22].

As seen in literature, these anomalies seem to be also evident in the craniofacial complexes of patients with certain malocclusions, as unilateral crossbites (Figure 10), lower midline deviations, and facial asymmetries. Because some asymmetric malocclusions are difficult to correct fully, it was hypothesized that generalized body asymmetry might underlie these malocclusions in some patients [17].

In a group of subjects controlled from April 2002 through July 2003, [23] the posteroanterior cephalometric radiographs and chest X-rays from 85 patients with jaw deformities and a control group of 20 patients with no jaw deformities were controlled. To measure the lateral shift of the mandible, a horizontal baseline (X axis) was drawn on the cephalogram connecting the intersection of the external margins of the orbits and the most lateral points of the greater wings of the sphenoid. A vertical baseline (Y axis) was then marked perpendicular to the × axis, intersecting the ethmoid crista galli. Then, the lateral displacement of the mandibular mentum from the Y axis was measured. Displacement to the right was designated positive; that to the left was designated negative. Cobb's method was used to measure scoliosis curves on chest X-rays; the direction of the curve was designated similarly. Of the 85 patients with jaw deformity, [23] (27.1%) had a Cobb angle exceeding 10°. None of the control group had scoliosis exceeding 10°. No correlation was found between the direction of mandibular displacement and the direction of scoliosis.

In 2008, in the analysis of 28 children with scoliosis (mean age: 14.7 y; SD: 2.3 y) compared with a control group of 68 orthopedically healthy children (mean age: 14.8 y; SD: 0.11 y), three children in the scoliotic group were registered with a unilateral crossbite (Figure 8) while there was only a bilateral crossbite case. In the control group two bilateral crossbite cases matched the three unilateral crossbite cases [18].

In the same sample, also the degree of the midline deviation (Figure 4), that is a clinical sign associated with the unilateral crossbite, was recorded. The severe type of scoliotic group was characterized by significantly higher prevalence of midline deviation, compared to the control group [18]. Also, this scoliotic group was found to have higher midline deviation values respect to the control group (Table 2).

In 2006, in a group of 96 orthopedic patients with idiopathic scoliosis 17 (79 females and 17 males: mean age, 13.9 y; SD: 3.5 y; range, 6.2-25.3 y), compared with a control group of 705 children, the prevalence of upper midline deviation was 21% in the group of scoliosis and 9.5% in the control group; while the prevalence of lower midline deviation was 53.7% in the study group and 32.9% in the control group. In the same sample, the prevalence of unilateral posterior crossbite was 28.1% and 18.1% respectively in the study and the control group; the prevalence of anterior crossbite was 16.6% in the study group and 9.3% in the control group.

An increased occurrence of orthopaedic alterations in the frontal plane was also observed in children with a unilateral crossbite in another recent study [24]. More specifically, among the children who revealed an asymmetric upper cervical spine, the unilateral crossbite was not necessarily combined with a pathological orthopaedic variable, but statistically, children with a unilateral crossbite showed more often an oblique shoulder, scoliosis, an oblique pelvis, and a functional leg length difference than children with symmetry. No correlation was found between the laterality of the crossbite side and any orthopaedic asymmetry. The study was conducted comparing fifty-five children aged 3-10 years (22 girls and 33 boys) with a unilateral crossbite and 55 gender- and age-matched children with asymmetric upper cervical spine, but no crossbite, who served as the control group, selected from an orthopaedic cohort of 240 patients. The certain asymmetry of the upper cervical region was confirmed in all the subjects by radiographs and palpation.

Also, in 2008, in the analysis of 28 children with scoliosis (mean age: 14.7 y; SD: 2.3 y) compared with a control group of 68 orthopedically healthy children (mean age: 14.8 y; SD: 0.11 y), 18 the clinical examination of the Temporo-mandibular joint (TMJ) at almost a quarter of the scoliotic group revealed a pathological symptom: the mandibular lateral movements showed a reduced range in only one side. More specifically, only half of the patients in the scoliotic group were able to make the same range of bilateral movements. On the contrary, this rate was 82.32% in the control group.

The results of all these cited studies suggest that dental asymmetries correlate with orthopaedic asymmetries in the frontal plane, when the analysis is conducted in a sample of young boys and girls.

However, it must be noted that also the bilateral crossbite was related to the scoliosis. In 2007, also a correlation between scoliosis and bilateral crossbite was reported: in a study on 428 subjects (211 females and 217 males), aged 9 to 14 years, 25 a 2.8% scoliosis incidence has emerged, and an incidence of scoliotic attitudes of 9.5%, with a statistically significant relationship among that disorders of posture, and the presence of ogival palate with bilateral crossbite.

Among the studies on the association between asymmetric occlusion and trunk asymmetry, a few studies must be cited, that have investigated these co-relationship in health subjects without any pathological orthopaedic condition [25-28]. They reported deviating findings: Lippold et al. (2000) [25] found a statistically significant correlation between midline deviation and oblique pelvis as well as leg length differences, considered in the limits of physiology. The other two studies [26,27] showed that moderate trunk asymmetry (not pathological condition) did not affect facial asymmetry or vice versa. With regard to the study design and the investigated patients, the three studies can hardly be compared: one [26] compared 29 children with a right-sided midline shift with 28 children with a symmetric occlusion; Lippold et al. (2000) [25] investigated midline discrepancies in 50 patients, aged 4-55 y, who were recruited from physiotherapy appointments, while Zepa et al. (2003) [27] analyzed frontal cephalograms and compared them with rib hump or lumbar prominence and spinal posture.

In order to investigate the possible effects of orthopaedic asymmetric disorders on dentofacial development and head posture, other clinical studies have been previously conducted on patients with scoliosis, and the results given by this previous literature are very similar to the more recent cited studies.

In general, in the previous literature on this field, the statistically recorded prevalence of unilateral crossbites in subjects with scoliosis amounted to 26-55 per cent [28-31].

Prager (1980) [30] interpreted the crossbite as a transmission of the asymmetry of the body, whereas Hirschfelder and Hirschfelder (1983) [32] considered, although they had not yet clarified transmission, the crossbite to be a new compensatory curvature of a scoliosis. Independent of the different offered explanations of the high prevalence of crossbite in those patients, an interdisciplinary treatment approach to alleviate facial asymmetry and to stabilize head posture, initiated as early as possible, has been unanimously recommended since '60 decade [28,33,34].

But it must be underlined that several studies about the association between unilateral crossbite and scoliosis were also conducted on animals, and the obtained results tended to confirm the observations recorded on humans.

In general, the results from experimental animal studies suggested that alterations in the occlusion evoke changes in many other regions of the body [35-37].

**Figure 11:**
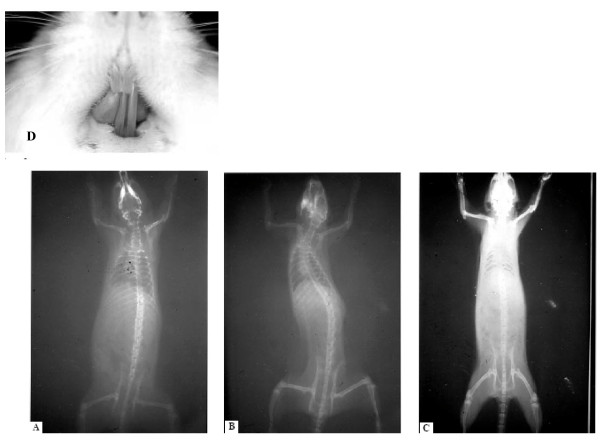
**(a) Before occlusal imbalance; (b) one week after occlusal imbalance; (c) one week after the balancing of occlusion; (d) occlusal imbalance through an unilateral crossbite**. Tracted by the paper referenced in [35,56].

For example, teeth occlusion seems to have an impact on head position, spinal column alignment, and masticatory muscles which control posture and modulate cardiac function via the trigeminal system. After unilateral occlusal destruction, a postural abnormality in terms of inability of head maintenance, T-wave inversion on electrocardiograph, hair loss, changes in tongue mobility, and eating disorders as well as pathologies of the eye have been observed [35]. Then, a scoliotic curve has been developed after induction of a unilateral crossbite in rats [34,36]. In these studies, the evoked changes were observed within 1 week of unilateral manipulation and normalized after harmonization of the occlusal plane (Figure 11)

Experimental studies also tried to explain the origin and the mechanism for the occurrence of an asymmetric growth of the head due to a unilateral crossbite: [38] more specifically, this experimental study in rabbits revealed a high level of asymmetry in craniofacial structures, temporomandibular structures and muscle functions in rabbits after an experimentally induced crossbite [37]. Moreover, in patients with a unilateral malocclusion, asymmetric condylar position with an asymmetric condylar path was observed, [28,39] and this seems to reduce the mandibular condylar growth, [40] causing an asymmetric mandibular ramus length, that has been observed shorter in the crossbite side [41,42]. Based on the findings that asymmetric facial structures can be corrected only after early correction of a unilateral crossbite [28,41,43] it was suggested that a persisting asymmetric occlusion results in growth restriction that leads to mandibular and facial asymmetry [40,44-47] and later also to a vertebral column asymmetry. For this type of correlation, the role of the cervical column, as a link tract between the head and the vertebral column has been underlined.

In this field, it has been demonstrated in health subjects, without evident orthopaedic disorders, that craniofacial growth is strongly associated with cervicovertebral anatomy [29,48]. It has been shown that the upper cervical region reveals a high potential for adaptation to craniofacial growth [29]. This may possibly be due to its important role: the cervical spine provides the morphological basis for an extensive freedom of head movement; then, it serves as a bridge for numerous blood and lymphatic vessels and nerves, linking head, trunk, and upper limb. The mechanism of transmission of an imbalance from the occlusion to the vertebral column may be related to the consequential tilt of the first cervical vertebra that affects the tilt of the adjacent vertebra, so destabilizing the vertical alignment of the cervical spine, also changing the functionality of each cervical muscle; finally, the asymmetrical distribution of loads could then affect the orientation of the other dorsal and lombar vertebrae, contributing to the functional deformity of the spine, finally the scoliosis [49].

Also, a close relationship among the masticatory muscles and the cervical muscles supporting the head has been demonstrated in patients requiring stomatognathic treatment [50]. In addition, it has been shown that occlusal interference can cause dysfunction of both the cervical spine and the sacro-iliac joint [51]. Consequently, all these authors recommended that the cervical spine and lumbar and pelvic regions should also be investigated in patients with craniomandibular dysfunction. In this field, the intimate developmental relationship between the atlas and the cranial base was also underlined [52].

In a study previously cited, [23] in facts, an oblique shoulder was diagnosed in 30.9% of the total group, and in 70.6% of them a unilateral crossbite was observed, suggesting a link among occlusion, cervical spine adaption and occurrence of scoliosis, although no causal relationship was demonstrated.

All the interdisciplinary studies on scoliotic patients are in accordance with the fact that no lateral correlation exists between the side of crossbite and the side of the curvature of the scoliotic spine [30,31,49].

Finally, in the analysis of the orthopedic literature, Floman [53] indicated a possible connection between thoracic scoliosis and restricted head motion in a report of 6 patients. However, it has not been clarified whether such a restriction in head motion had a secondary influence on occlusion.

## Discussion and conclusion

As seen, no randomized clinical trials were recorded. The observations were mostly based on case-control studies and clinical case-reports.

Longitudinal cllinical trials with a control group evidenced the association betweeh the first type of brace and the occurrence of a class II relationship induced by the brace; consequently, the clinical controlled trial suggested the use on orthodontic treatment during the treatment of scoliosis with a brace.

The maiority of other studies were case-control studies that evidenced the presence of unilateral class II, midline deviation, increased overjet and unilateral cross-bite in a higher percentage in subjects with scoliosis respect to health subjects.

As seen in this review, there are only few articles which describe the orthodontic examination as an opportunity for the early detection of scoliosis or which emphasize the necessity of early orthodontic check-ups for children with diagnosed scoliosis, highlighting the application of minimal-invasive methods of screening the affected population [54]. Based on their results, however, a dominancy of the dentofacial asymmetry (mostly unilateral crossbite) in the scoliotic group, can be expected, [48,55] as well as unilateral Angle class II malocclusion and midline deviation. As seen in this review, the data in literature prove also the existence of other dentofacial anomalies in children with scoliosis (ogival palate, increased overjet, reduced overbite, reduced range in lateral movements in one side), although these studies did not analyzed the orthopaedic sample on the base of the scoliotic angle, or the presence of one or more curves, and on the location of the curvature, which may affect the gravity and the type of scoliosis.

Some hypothesis to explain the correlation between the occlusion and the scoliosis can be made, although without certain conclusions.

The assumption on which the hypothesis on this correlation is based is that there is an anatomical and functional relationship between the stomatognathic apparatus and the spinal column. This relationship was hypothesized by several authors, based upon various observations [56].

Neurophysiological principles of convergence and sensitization: a constant input, such as a nociceptive input, on second-order neurons may increase the sensitivity of these neurons. Then, non-nociceptive neural impulses from other areas within the same segment, which converge onto these neurons, may give rise to altered sensations from these areas. For the craniocervical region, for example, a constant nociceptive input from, the upper part of the trapezius muscle can lead to an increased sensitivity of the spinal trigeminal nucleus and, consequently, non-nociceptive stimuli from the masticatory system would then lead to painful sensations from the trigeminal region [55]. This occurs as the different input converges onto the nucleus caudal portion of the trigeminal spinal tract nucleus [55]. As a consequence, for example, a significantly higher prevalence of cervical spinal pain was observed in a group of patients with craniomandibular pain than in a matched control group without craniomandibular pain, thus causing postural disease, and affecting in final the whole vertebral column.

2. Anatomical details: There is an anatomical relationship between the mandible and the cervical column, since the cranium and the mandible have muscular and ligament attachments to the cervical area. The function of the head, neck, and jaws is closely interrelated, forming a combined functional system [55,56]. observed a significant correlation between mandibular length and cervical lordosis angle on lateral skull radiographs (in natural head position) in Caucasian adult women with a skeletal class II malocclusion. The longer the mandibular body was, the straighter the cervical column appeared to be [56]. In a group of 50 Caucasian adult women with internal derangement, compared with a control group of 50 Caucasian women without internal derangement, cephalometric tracings on lateral skull radiographs in natural head position showed a significantly lower cervical lordosis angle. Beyond possibly causing TMJ diseases, dental malocclusions could, by the same mechanism, be linked to a functional asymmetry of trunk muscles. We suggest that one pathway is through the atlas. The atlas is linked to occipital condyles and thus affect the rest of the spine alignment, leading to further profound compensatory chenges, that may become pathological.

In conclusion, from a clinical point of view, if the asymmetry underlying idiopathic scoliosis and asymmetric malocclusion originates from the same etiology, it might be difficult to fully correct all features of the malocclusion or maintain the correction. This difficulty was observed, for example, in patients with posterior crossbites in whom relapse of lower midline deviations or tendency toward crossbites was evident also after orthodontic treatment. To clarify this point, the possibility of a connection between the reverse cycle in masticatory movements and asymmetrical posture should be evaluated.

In conclusion, all the observed frequent and severe dentofacial deviations in the scoliotic group draw the attention to the necessity of the early examination of this patient group from an orthodontics and orthopaedic point of view.

However, whether scoliosis affects mandibular dentoalveolar symmetry (whether there is a causal relationship) needs further elucidation.

## Competing interests

The authors declare that they have no competing interests.

## Authors' contributions

ST and MSare the Principal Investigator of this review. They 1) have made substantial contributions to conception and design, acquisition of data, analysis and interpretation of data; 2) have been involved in drafting the manuscript or revising it critically for important intellectual content; and 3) have given final approval of the version to be published. ST and MS contributed in the same way, as principal investigators, to this research. LT participated in drafting the manuscript. SM helped in the revision of the manuscript. AP helped in the first revision of the manuscript. FF participated in drafting the manuscript. All authors read and approved the final manuscript.
